# Coexpression enhances cross-species integration of single-cell RNA sequencing across diverse plant species

**DOI:** 10.1038/s41477-024-01738-4

**Published:** 2024-06-27

**Authors:** Michael John Passalacqua, Jesse Gillis

**Affiliations:** 1 Genomics Department, Cold Spring Harbor Laboratory, Cold Spring Harbor, NY USA; 2 Physiology Department and Donnelly Centre for Cellular and Biomolecular Research, University of Toronto, Toronto, Ontario Canada

**Keywords:** Transcriptomics, Plant evolution, Bioinformatics, Reverse transcription polymerase chain reaction, Software

## Abstract

Single-cell RNA sequencing is increasingly used to investigate cross-species differences driven by gene expression and cell-type composition in plants. However, the frequent expansion of plant gene families due to whole-genome duplications makes identification of one-to-one orthologues difficult, complicating integration. Here we demonstrate that coexpression can be used to trim many-to-many orthology families down to identify one-to-one gene pairs with proxy expression profiles, improving the performance of traditional integration methods and reducing barriers to integration across a diverse array of plant species.

## Abstract (web summary)

To enhance cross-species single-cell analysis, the authors find gene pairs with similar expression patterns across 13 species. These coexpression proxies serve as common features in datasets, improving integrative and comparative cell-type analysis.

## Main

Plants have a remarkably flexible cellular physiology, driving their adaptation into nearly every environment. Recently, the advent of single-cell RNA sequencing (scRNA-seq) has provided novel insights into the diversity of cell types underlying these adaptations^1,2^. The unique diversity in plants makes comparative assessments between species important but is complicated by uncertain homology relationships. Unlike in mammals, where homologous genes and structures can be easily identified, plant gene families frequently expand by whole-genome duplication, polyploidization and tandem gene duplication^3–5^. This scarcity of one-to-one gene pairs is a major barrier to defining a common gene space for the integration of single-cell data, a key step for successful cross-species comparative analysis or integration^6,7^. With vast amounts of plant scRNA-seq data becoming available^8^, this study aims to address a critical gap in its analysis by using coexpression to identify pairs of genes that, while not exclusive orthologues, are functionally related enough to enable the integration of this high-dimensional data. By reducing barriers to integration, we prime the field for the discovery of novel, cell-type specific innovations that have been critical to plant adaptation and domestication.

**Fig. 1:**
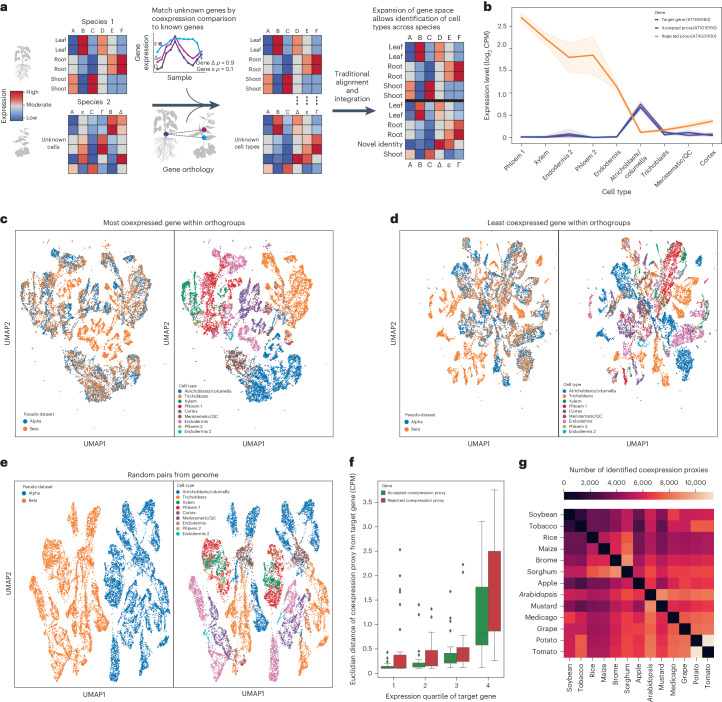
Coexpression proxies integrate a split dataset without shared genes. **a**, Schematic depicting the identification of coexpression proxies from gene orthology information and their use in expanding the gene space to enable integration followed by identification of novel and conserved cell types. **b**, Gene expression profile for target gene (*AT1G16160*) and two potential coexpression proxies (*AT1G16150*, *AT4G31100*). The gene with the more similar profile, *AT1G16150*, was identified as a coexpression proxy, while *AT4G31100* was rejected. The centre band is the mean counts per million (CPM) for each gene in the cell type in our single-cell dataset. The error bar is the 95% confidence interval. QC, quiescent center. **c**, UMAP showing integration of a split and dissociated *A. thaliana* dataset containing 16,636 cells using coexpression proxies. **d**, UMAP showing integration of the same dataset using the worst potential coexpression proxy from each gene family. **e**, UMAP showing the failed integration of the split and dissociated dataset using 1,900 random gene pairs. **f**, Euclidian distance from the expression profile of the target gene for *n* = 117 pairs of accepted coexpression proxies and rejected coexpression proxies in independent cell types, split by expression quartile of the target gene. The bottom of the box is the lower quartile, the top of the box is the upper quartile and the centre bar is the median. The whiskers are 1.5 times the interquartile range. **g**, Heat map showing the number of identified coexpression proxies between each species pair in the database.

While a given plant sample may have thousands of expressed genes, the expression patterns of these genes are not independent and are instead organized into the regulatory programs that underlie cell types. This coexpression generates the low-dimensional expression space that is foundational to the success of modern single-cell analysis^9^. We hypothesize that genes with highly similar expression profiles between two species can be used as reasonable proxies for integrating cell-type specific data, that we can identify such profiles using coexpression and that this will expand the shared gene space, improving our ability to compare cross-species data. The essence of the approach is to use meta-analysis from previous bulk RNA sequencing data to define cross-species gene pairs (coexpression proxies) that can be applied to more specific, but sparser, single-cell data. By utilizing robust coexpression networks built from over 16,000 publicly available RNA sequencing datasets, as well as gene phylogenies from OrthoDB v11 (a database of precomputed gene orthology relationships), we ensure that the coexpression proxies accurately reflect the underlying biology of each species pair they are drawn from^10,11^. We illustrate this approach, where coexpression data and gene phylogenies identify gene pairs that expand the one-to-one (1–1) gene space, improving data integration and alignment between known cell types and highlighting novel ones between species (Fig. 1a). While previous work has expanded the shared gene space through gene homology comparisons, our focus on coexpression uniquely captures both regulatory and functional shifts between species^12^. By improving integration, we enable researchers to identify new and conserved cell types in their scRNA-seq data. We validate the coexpression proxies with two test examples, highlighting their utility. In the first test, we show that coexpression proxies can accurately reintegrate a split dataset with no shared gene space. Second, we show that coexpression proxies improve the integration of real single-cell data between two species with complex genomes: maize and rice.

Our first test is an extreme one in which we generate and integrate two cross-species datasets with no one-to-one orthologues. This would be impossible with a traditional integration approach, which requires directly matched one-to-one orthology relationships between genes in each species for alignment before integration. To construct a case with a ground truth integration without using synthetic data, we split an existing *Arabidopsis* single-cell dataset into two pseudo-‘species’. The first ‘species’ is generated by randomly selecting half of the cells as well as half the genome. For these cells, the second half of the genome is removed. We then take the remaining cells, which will become our second ‘species’, and remove the half of the genome present in the first set of cells (Supplementary Fig. 1). This provides two sets of cells with known, shared cell types and distinct genomes. We then identify coexpression proxies between the two subset genomes, finding pairs of genes with similar expression profiles. As an example, the selected coexpression proxy gene, *AT1G16150*, closely matches the expression profile of the target gene, *AT1G16160*. By contrast, *AT4G31100*, a rejected gene from the same orthologue family, has a distinct expression profile (Fig. 1b).

Next, we used these coexpression proxies to reintegrate the split *Arabidopsis* dataset. Highlighting that coexpression proxies smoothly integrate into existing workflows, we used Scanorama v1.7.1^13^ to reintegrate and re-cluster the dataset, placing 82% of cells into a cluster with cells from both datasets (Fig. 1c). The reintegration was accurate, successfully matching cells of the same cell type across datasets 75% of the time. To evaluate how much of the gene proxies’ success was dependent on information from the gene phylogenies and how much information was derived from the coexpression conservation profile, we attempted to integrate the datasets using the worst rejected proxy from within each orthologue group (that is, the proxy with the lowest coexpression). Performance was lower using these gene pairs, reducing the successful matching of cells to 65% (Fig. 1d). This moderate performance suggests that simple relaxation of orthology constraints is a substantial contributor to performance. However, coexpression provides a substantial overall signal boost. This was particularly clear for phloem, which was otherwise unintegrated or mixed with atrichoblasts and xylem. To determine whether sequence similarity alone would prove sufficient, we calculated the pairwise protein sequence similarity of every *Arabidopsis* gene and attempted to use this to identify gene proxies. While able to perform better than random, this metric was worse than coexpression at reintegrating the split dataset and completely failed to reintegrate certain clusters. Finally, we attempted integration using 1,900 random gene pairs and found that we were unable to achieve any integration (Fig. 1e). To further evaluate our coexpression proxies, we assessed the degree to which rejected and selected gene pairs show the same expression across cell types on a per-gene basis (measured by Euclidean distance). We found that accepted coexpression proxies are much closer to the target’s expression profile across cell types and that the rejected proxies are on average 83% further from the target’s expression (Fig. 1f). This shows that the coexpression proxies are more similar in expression profile to their target genes than even other genes from the same orthogroup.

Given the success of our approach, we generated coexpression proxies between 13 plant species and identified an average of 5,750 gene pairs between species (Fig. 1g). The coexpression proxies are numerous enough to provide additional information across even highly diverged species and are well represented (4,899 pairs) even between *Zea mays* and *Arabidopsis thaliana*, which diverged 160 Ma. Importantly, although we used Scanorama, these coexpression proxies can be easily incorporated into any potential integration pipeline as they simply expand the shared feature space.

**Fig. 2:**
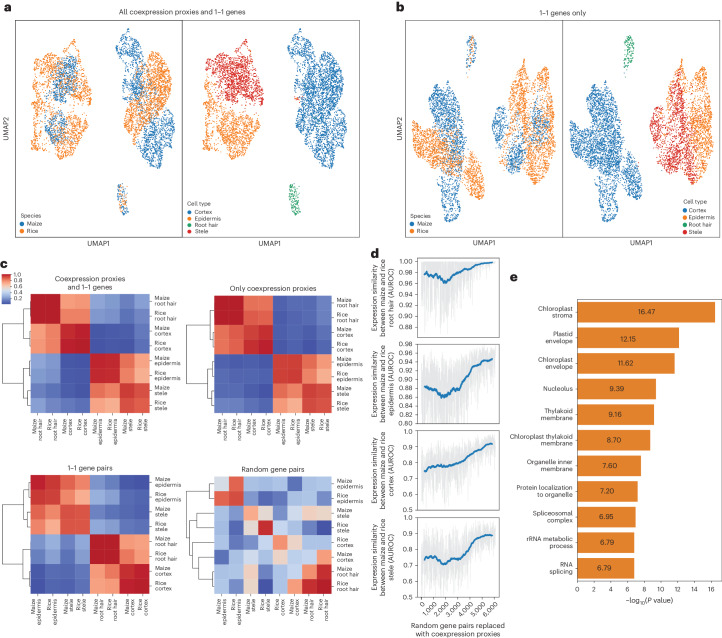
Integration of maize and rice scRNA-seq data using coexpression proxies. **a**, UMAP showing integration of 2,832 *Z. mays* cells and 3,500 *Oryza sativa* cells using coexpression proxies. **b**, UMAP showing integration of *Z. mays* and *O. sativa* using only 1–1 gene pairs from OrthoDB. **c**, MetaNeighbor plots showing post integration similarity between cell types using four different gene sets. **d**, Improvement in integration across 872 integration runs as random gene pairs are gradually swapped for coexpression proxies. **e**, Enriched Gene Ontology terms among rice–maize coexpression proxies. Enrichment was tested by a two-sided Fisher’s exact test. It was then corrected using the Benjamini–Hochberg correction.

Having shown that coexpression proxies could integrate an otherwise uncorrectable dataset, we tested their ability to improve the integration of single-cell data across two different species. Using a supervised integration, we attempted the integration of two root datasets, one from maize and one from rice. We focused on four broad cell types for which author annotations directly aligned. Using coexpression proxies, we successfully integrated the maize and rice dataset, accurately integrating 36% of cells into clusters with cells from both datasets (Fig. 2a and Supplementary Fig. 2). The remaining cells were different enough to still appear as distinct sub-clusters across species. While this is far from 100%, real cross-species differences do exist, so it is not clear what the maximum plausible integration percentage is. Importantly, our integration is better than using only the 1–1 gene pairs, which integrated only 14% of the cells (Fig. 2b). Key cell types, such as epidermis and stele, are well integrated using coexpression and are less well integrated by 1–1 gene pairs, as evidenced by lack of species mixing within cell types and close proximity across cell types. Similarly, coexpression did not overfit away real differences, capturing the likely real difference between cortex cells where constitutive aerenchyma formation is critical to oxygen diffusion in partially submerged rice^14^. To evaluate the integration on a cell-type-by-cell-type basis, we used MetaNeighbor v3.19, which enables us to quantify the degree to which cell types replicate across datasets in a statistical framework^15,16^. We compare four integrations using scGen—utilizing coexpression proxies and 1–1 genes, using only coexpression proxies, using only 1–1 genes and using random genes (Fig. 2c). As can be seen, coexpression proxies alone, 1–1 pairs alone and the combination all accurately and similarly group cell types across species. While subtle for this broad classification, the full coexpression proxy set integrates better than either of its parts in all cell types when evaluated by MetaNeighbor (except cortex, where all methods are perfect), reflecting the additional information from the coexpression proxies. Because this is a validation focused on well-defined alignment, performances generally go from high to even higher (for example, stele goes from AUROC (area under the receiver operator curve) 0.93 to 0.973). To evaluate the utility of an increased known gene-pair space, as well as the robustness of the model, we swapped in coexpression proxies for random pairs and tracked performance improvement (Fig. 2d). Performance increases steadily to near 1 for most cell types, indicating that the typical number of 5,000 coexpression proxies is sufficient to integrate cross-species data. Further querying the coexpression proxies, we found they typically represented core conserved functions such as photosynthesis, mitochondrial proteins and ribosome metabolism (Fig. 2e).

Integrating cross-species single-cell data is an increasingly common goal in the fields of plant development, evolution and molecular biology. To facilitate this process, we have demonstrated that using coexpression proxies expands the gene space available for integration. To facilitate adoption of this approach by the community, we have generated pairwise coexpression proxies between 13 plant species at 3 thresholds. All coexpression proxy lists have been made available at https://gillislab.shinyapps.io/epiphites_v11/. In addition, we have provided a workflow for generating a coexpression network from scRNA-seq data and using it to identify coexpression proxies for integration (Supplementary Code), which additionally requires only gene phylogenies between the two species. We show that this approach generates networks similar to gold standard networks and enables similar integration (Supplementary Figs. 3 and 4). These proxy lists provide an important resource for improving the integration of single-cell data, accelerating the transfer of knowledge from well-studied model organisms to crop systems that are crucial to the global food supply.

## Methods

### Gene coexpression proxy identification

For each species pair, gene family orthology information was downloaded from OrthoDB V11^11^. Utilizing one-to-one gene pairs, coexpression conservation was calculated between all genes in each species^17^. Briefly, we compare each gene’s top 10 coexpression partners across species. These top 10 are limited to genes that are one-to-one orthologues, although the matching of proxies is not limited in this way. Using one-to-ones as a basis set for comparison of other genes expands the range of potential proxies while still leaving it grounded in defined cross-species overlaps. We use the ranks from one species to predict the coexpression partners of the second species and then repeat this in the other direction, averaging the scores to generate the conservation of coexpression score, which is an AUROC. This resulted in a species A genes by species B genes matrix, filled with the AUROC score for each gene pair. For each gene family, the coexpression conservation matrix was filtered to every possible cross-species gene pair. Next, pairs in multigene groups were eliminated by thresholding in two steps. First, any gene pairs with scores below a quality threshold were discarded. Second, remaining pairs were required to be reciprocal best hits and to be higher than other potential options by a multi-pair threshold. For genes that were one-to-one matches, they were only discarded if below a lower single pair quality threshold. For the moderate filtering, the quality, multi-pair and single-pair junk thresholds were 0.85, 0.03 and 0.8. For lenient filtering, the thresholds were 0.8, 0.02 and 0.7, and for stringent filtering, these were 0.9, 0.035 and 0.85. The moderate threshold was chosen by evaluating the number of proxies identified at many thresholds and choosing the elbow, and lenient and stringent thresholds were picked to form a 0.1 range around this number.

### Dataset integration and evaluation

To generate an integration task that was uncorrectable without a shared gene space, the *Arabidopsis* dataset was split into two sets of cells. Using Pandas DataFrame.sample, one half of the genome was randomly selected and assigned to the first set of cells, with other data being discarded. The second set of cells were assigned the second half of the genome, and the genes assigned to the first half were discarded. Utilizing the same method as above, coexpression proxies were identified between the two halves of the genome with the moderate threshold. Aligning the two gene spaces using these proxies, we performed integration using the Scanorama v1.7.1 Python package^13^. The scanorama.integrate function was used to integrate the two datasets into a shared low-dimensional space, and this was plotted using scanpy.pp.neighbors with the default parameters (15 nearest neighbours, 50 principal components) and the scanpy.tl.umap function (default parameters). For evaluation, we first clustered the integrated data using scanpy.tl.leiden at a resolution of 0.5. This provided an evaluation space that is based on the high-dimensional underlying data, instead of the 2D uniform manifold approximation and projection (UMAP), which can be misleading. Then, using these clusters, we defined a cluster of the same cell type as one containing more than 60% of that cell type and a mixed cluster as one composed of between 30% and 70% of each starting dataset. In plotted boxplots, the centre line is the median, the box limits are the upper and lower quartiles, the whiskers are 1.5 times the interquartile range and the points are any datapoints beyond the whisker range.

As the cross-species integration scenario was more challenging, it was integrated utilizing scGEN v2.1.0^18^. The datasets were limited to 4 broad cell types for which author annotations clearly aligned, and the rice dataset was subset to 3,500 cells to match the maize dataset of 2,832 cells. The tissues were equally represented in each of the two datasets. Utilizing coexpression proxies between rice and maize at the moderate threshold, the two datasets were aligned. The two datasets were first aligned utilizing coexpression proxies between rice and maize at the moderate threshold. Next, the scGEN model was initialized using scgen.SCGEN and trained using scgen.model.train, using default parameters. Next, the integration was performed using scgen.model.batch_removal. To evaluate the integration beyond the low-dimensional representation, MetaNeighbor was used to compare the post-integration similarity of cell types^15^. To confirm the model was utilizing coexpression proxies and not relying on training information, the integration was run 872 times, starting with random gene pairs. Following each run, seven random pairs were replaced with seven coexpression proxies, until all were replaced. Gene Ontology term enrichment was performed using Fisher’s exact test from scipy.stats.fisher_exact() to find terms over-represented in the coexpression proxies, utilizing all genes in the bulk network as the background gene set. Multiple hypothesis correction was performed using the Benjamini–Hochberg correction function from statsmodels.stats.multitest.multipletests() at alpha .05.

### Reporting summary

Further information on research design is available in the Nature Portfolio Reporting Summary linked to this article.

### Supplementary information

Supplementary Information Supplementary Figs. 1–4.

Reporting Summary

Supplementary Code Explained code and workflow to generate a coexpression network from a single-cell data file.

## Data Availability

All analyses were performed in Python v3.9, Pandas v1.5, SCGEN v2.1.0, Statsmodels v0.14.1, Scipy v1.12.0, Scanorama v1.7.1 and SCANPY v1.9.1^19^. Aggregate coexpression networks were downloaded from CoCoCoNet^10^. *A. thaliana* single-cell RNA-seq expression data totalling 16,636 cells from 4 datasets were downloaded from the Gene Expression Omnibus (GEO IDs: GSE116614, GSE121619, GSE123818, GSE123013)^1,2,20,21^. Cluster assignments were downloaded from GEO for IDs GSE121619 and GSE123013 or provided by the authors for IDs GSE123981 and GSE116614. *O. sativa* single-cell RNA-seq expression data and accompanying cluster assignments were downloaded from GSE146035^22^. *Z. mays* single-cell RNA-seq expression data and accompanying cluster assignments were downloaded from GSE183171^23^, and only nitrate-treated cells were used. Orthology information is from OrthoDB V11 (https://www.orthodb.org/).

## Code availability

All code is available via our repository at https://github.com/gillislab/Coexpression_Proxies.
