# Supplementary material for: Coexpression enhances cross-species integration of single-cell RNA sequencing across diverse plant species
Source: Nat Plants. 2024 Jun 27;10(7):1075–80. doi: 10.1038/s41477-024-01738-4 (PMC11266096; doi:10.1038/s41477-024-01738-4)
Supplement: Supplementary file 1 — Supplementary Figs. 1–4. [file 41477_2024_1738_MOESM1_ESM.pdf]

nature plants

Supplementary information

https://doi.org/10.1038/s41477-024-01738-4

Coexpression enhances cross-species integration of single-cell RNA sequencing across diverse plant species

In the format provided by the authors and unedited

Supplemental Figure 1

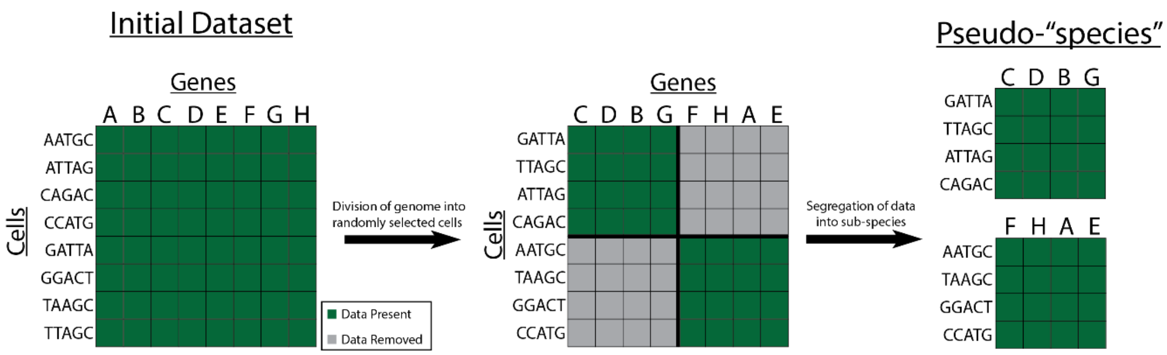

Supplementary Figure 1: Schematic illustrating how an initial dataset consisting of cells from one species is split into two datasets with no shared genes or cells, generating two "species" with a ground truth of shared cell types.

Supplemental Figure 2

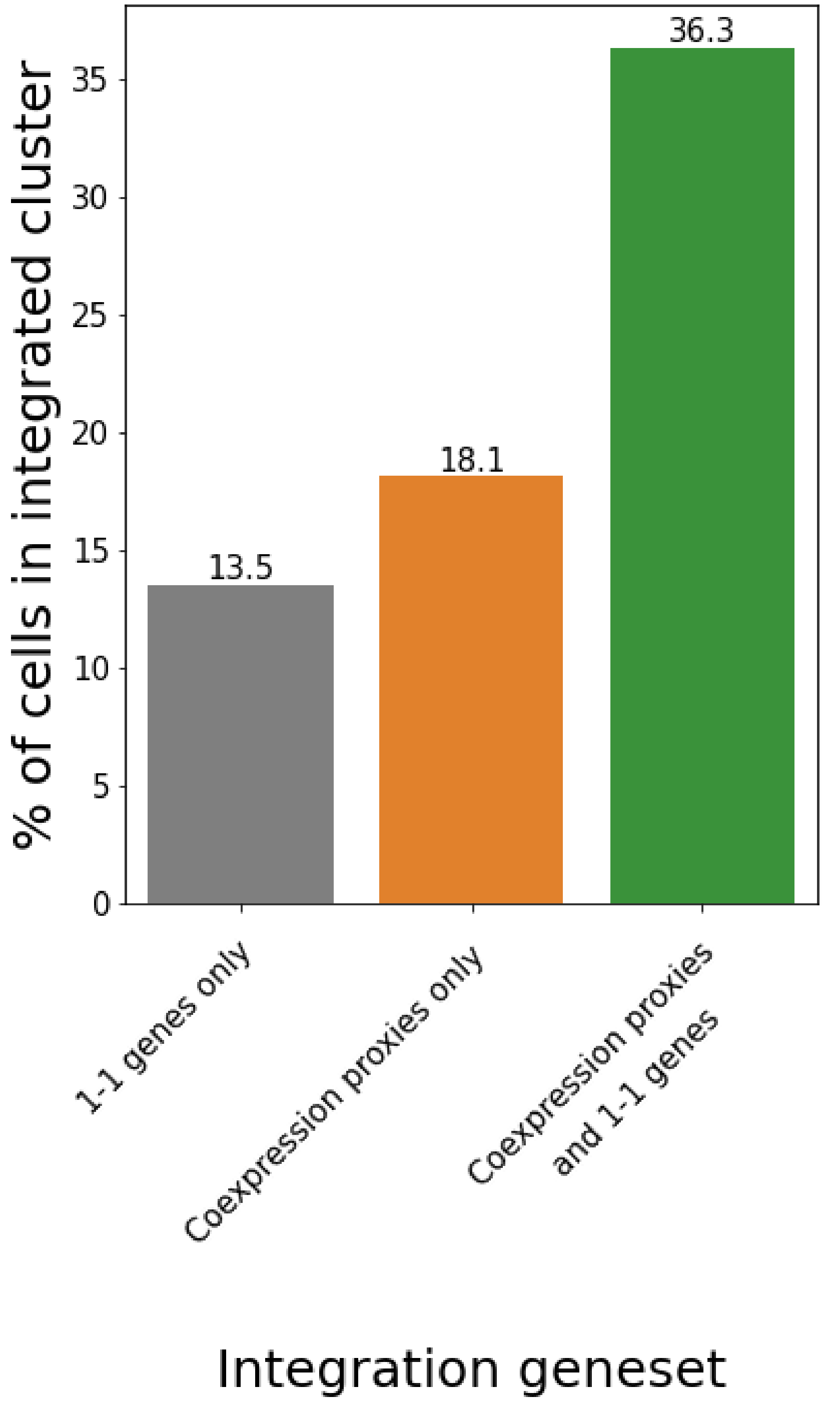

Supplemental Figure 2: Bar chart showing the percentage of maize and rice cells in a cluster with either dataset comprising no more that 70% of the cluster.

Supplemental Figure 3

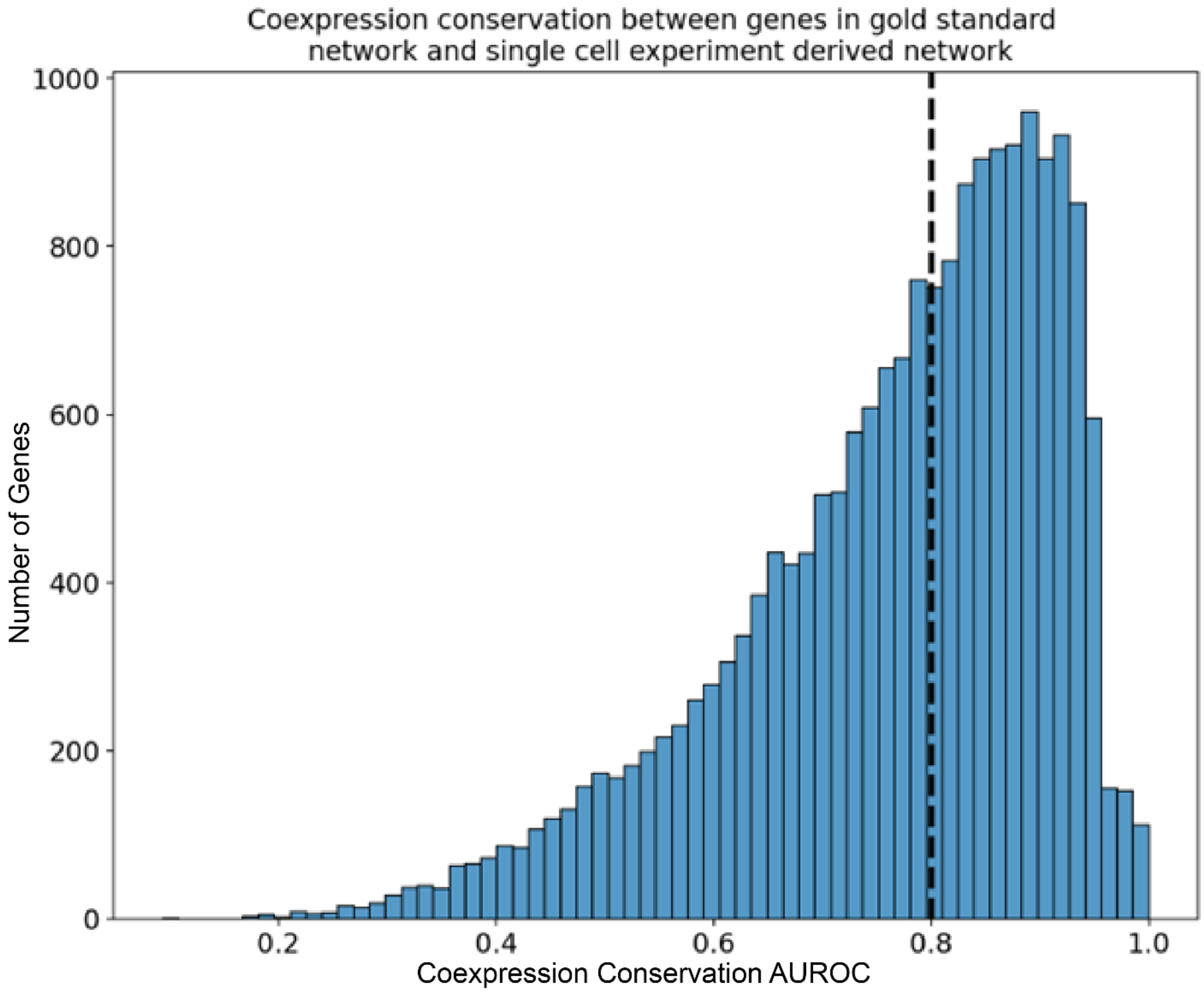

Supplemental Figure 3: Distribution of coexpression conservation scores for every *Arabidopsis thaliana* gene between the existing gold standard network and a new coexpression network generated using our workflow on *Arabidopsis thaliana* root cell data.

Supplemental Figure 4

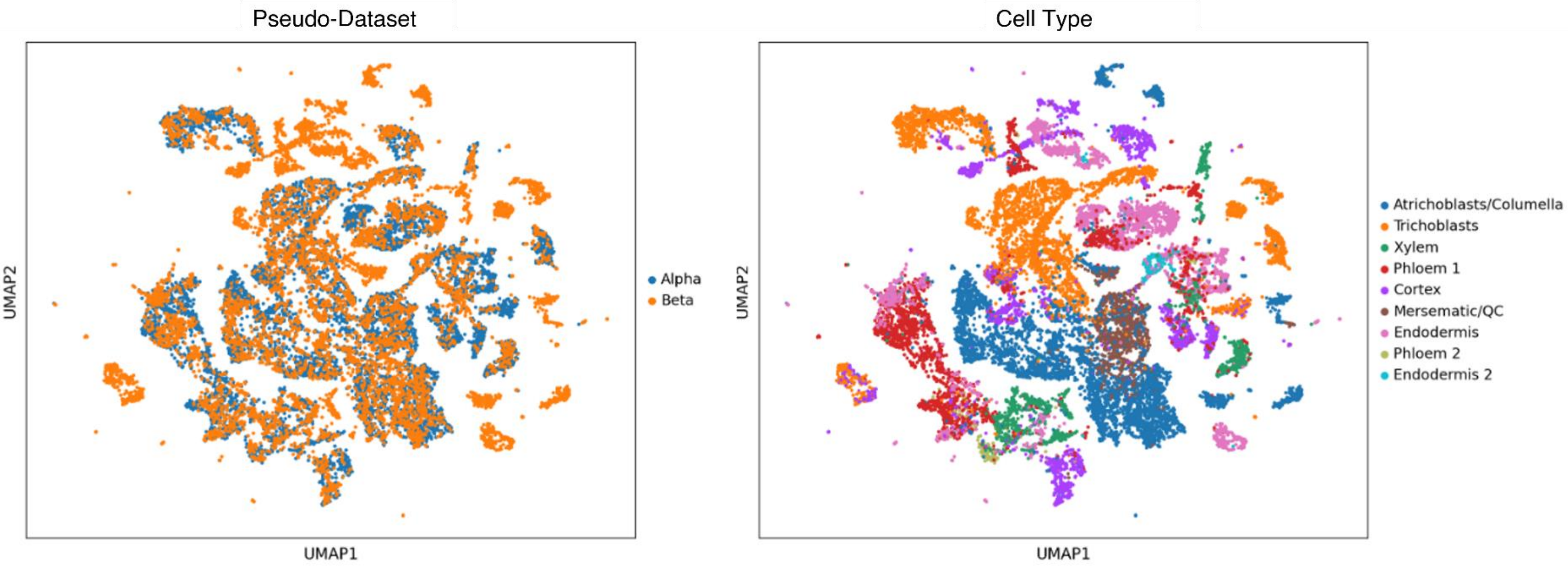

Supplemental Figure 4: UMAP showing integration of a split and disassociated Arabidopsis thaliana dataset containing 16636 cells. The alpha dataset was assigned the existing gold standard coexpression network, and the beta dataset was assigned a new coexpression network built using our workflow for scRNA-seq data, and 1293 coexpression proxies were identified between the datasets for integration.
