# Supplementary material for: Coexpression enhances cross-species integration of single-cell RNA sequencing across diverse plant species
Source: Nat Plants. 2024 Jun 27;10(7):1075–80. doi: 10.1038/s41477-024-01738-4 (PMC11266096; doi:10.1038/s41477-024-01738-4)
Supplement: Supplementary file 2 — Reporting Summary [file 41477_2024_1738_MOESM2_ESM.pdf]

nature portfolio

Corresponding author(s): Jesse Gillis

Last updated by author(s): 5/3/2024

Reporting Summary

Nature Portfolio wishes to improve the reproducibility of the work that we publish. This form provides structure for consistency and transparency in reporting. For further information on Nature Portfolio policies, see our Editorial Policies and the Editorial Policy Checklist.

Statistics

For all statistical analyses, confirm that the following items are present in the figure legend, table legend, main text, or Methods section.

n/a	Confirmed	
☐	☒	The exact sample size (*n*) for each experimental group/condition, given as a discrete number and unit of measurement
☒	☐	A statement on whether measurements were taken from distinct samples or whether the same sample was measured repeatedly
☐	☒	The statistical test(s) used AND whether they are one- or two-sided<br>*Only common tests should be described solely by name; describe more complex techniques in the Methods section.*
☒	☐	A description of all covariates tested
☐	☒	A description of any assumptions or corrections, such as tests of normality and adjustment for multiple comparisons
☐	☒	A full description of the statistical parameters including central tendency (e.g. means) or other basic estimates (e.g. regression coefficient) AND variation (e.g. standard deviation) or associated estimates of uncertainty (e.g. confidence intervals)
☐	☒	For null hypothesis testing, the test statistic (e.g. *F*, *t*, *r*) with confidence intervals, effect sizes, degrees of freedom and *P* value noted<br>*Give P values as exact values whenever suitable.*
☒	☐	For Bayesian analysis, information on the choice of priors and Markov chain Monte Carlo settings
☒	☐	For hierarchical and complex designs, identification of the appropriate level for tests and full reporting of outcomes
☒	☐	Estimates of effect sizes (e.g. Cohen's *d*, Pearson's *r*), indicating how they were calculated

Our web collection on statistics for biologists contains articles on many of the points above.

Software and code

Policy information about availability of computer code

Data collection	No software was used for data collection
Data analysis	All code used is available on our repository at https://github.com/gillislab/Coexpression_Proxies<br>All analyses were performed in Python 3.9, Pandas 1.5,SCGEN 2.1.0, Statsmodels 0.14.1, Scipy 1.12.0, and SCANPY version 1.9.117

For manuscripts utilizing custom algorithms or software that are central to the research but not yet described in published literature, software must be made available to editors and reviewers. We strongly encourage code deposition in a community repository (e.g. GitHub). See the Nature Portfolio guidelines for submitting code & software for further information.

Data

Policy information about availability of data

All manuscripts must include a data availability statement. This statement should provide the following information, where applicable:

- Accession codes, unique identifiers, or web links for publicly available datasets
- A description of any restrictions on data availability
- For clinical datasets or third party data, please ensure that the statement adheres to our policy

Aggregate coexpression networks were downloaded from CoCoCoNet10. Arabidopsis thaliana single-cell RNA-seq expression data totaling 16636 cells from 4 datasets were downloaded from the Gene Expression Omnibus GEO IDs: GSE116614, GSE121619, GSE123818, GSE123013)1,2,18,19. Cluster assignments were downloaded from GEO for IDs GSE121619 and GSE123013, or provided by the authors for IDs GSE123981 and GSE116614. Oryza sativa single-cell RNA-seq

expression data and accompanying cluster assignments were downloaded from GSE14603520. Zea mays single-cell RNA-seq expression data and accompanying cluster assignments were downloaded from GSE18317121, and only nitrate treated cells were used. Orthology information is from OrthoDBv11 - https://www.orthodb.org/

Research involving human participants, their data, or biological material

Policy information about studies with human participants or human data. See also policy information about sex, gender (identity/presentation), and sexual orientation and race, ethnicity and racism.

Reporting on sex and gender	No human related research has been used in this manuscript
Reporting on race, ethnicity, or other socially relevant groupings	No human related research has been used in this manuscript
Population characteristics	No human related research has been used in this manuscript
Recruitment	No human related research has been used in this manuscript
Ethics oversight	No human related research has been used in this manuscript

Note that full information on the approval of the study protocol must also be provided in the manuscript.

Field-specific reporting

Please select the one below that is the best fit for your research. If you are not sure, read the appropriate sections before making your selection.

☒ Life sciences ☐ Behavioural & social sciences ☐ Ecological, evolutionary & environmental sciences

For a reference copy of the document with all sections, see nature.com/documents/nr-reporting-summary-flat.pdf

Life sciences study design

All studies must disclose on these points even when the disclosure is negative.

Sample size	No sample size calculation was performed. We utilized available, existing scRNA sequencing plant data sets for testing, using all cells available for integration - which is how our sample size was chosen. We show that sufficient genes are used in Figure 2D
Data exclusions	Cell types without clear matches between maize and rice were excluded from the maize rice integration. These were predetermined for exclusion based on annotation.
Replication	All experiments were repeated with multiple random seeds to confirm robustness and confirm initialization did not impact results. All replication was successful. All experiments with non-deterministic code were repeated at minimum of 3 times. The maize rice integration was also repeated 872 times to confirm robustness of results as the number of available genes changed.
Randomization	Arabidopsis cells were randomly assigned to each psuedo data set. No other randomization was relevant to the study as there were no direct experiments.
Blinding	Blinding was not possible as there was only evaluation of existing data to identify co-expression proxies. Existing data had known ground truth cell types, and we only evaluated how well we could restore this known ground truth. There was nothing possible for us to blind.

Reporting for specific materials, systems and methods

We require information from authors about some types of materials, experimental systems and methods used in many studies. Here, indicate whether each material, system or method listed is relevant to your study. If you are not sure if a list item applies to your research, read the appropriate section before selecting a response.

Materials & experimental systems

n/a	Involved in the study
☒	☐ Antibodies
☒	☐ Eukaryotic cell lines
☒	☐ Palaeontology and archaeology
☒	☐ Animals and other organisms
☒	☐ Clinical data
☒	☐ Dual use research of concern
☐	☒ Plants

Methods

n/a	Involved in the study
☒	☐ ChIP-seq
☒	☐ Flow cytometry
☒	☐ MRI-based neuroimaging

Plants

Seed stocks	No seed stocks were used, all data was from publicly available data sets
Novel plant genotypes	No new genotypes were used, all data was from publicly available data sets
Authentication	No authentication was performed, as no new data was generated
